# preVIEW: COVID-19

**DOI:** 10.29173/jchla29667

**Published:** 2023-04-01

**Authors:** Sarah C. McGill

**Affiliations:** Research Information Specialist, CADTH (http://www.cadth.ca/), Ottawa, Ontario, Canada

**Product:** preVIEW: COVID-19

**URL:** https://preview.zbmed.de/

**Intended audience:** Anyone searching for COVID-19 preprints. Familiarity with subject headings and Boolean operators is helpful.

## Product Description

PreVIEW is a free, web-based COVID-19 preprint search engine combining seven preprint servers [1] and with over 66,000 preprints as of January 2023. It was developed by the German NFDI4Health Task Force COVID-19 (https://www.nfdi4health.de/en/task-force-covid-19.html), is funded by the German Research Foundation (the Deutsche Forschungsgemeinschaft (DFG) https://www.dfg.de/ ) [2], and is updated daily [1]. PreVIEW uses artificial intelligence (AI) to tag or annotate preprints with subject heading-like semantic concepts [1, 3, 4].

## Usability

### Searching

A search for a specific semantic concept in preVIEW, such as the Omicron variant, will retrieve all preprints annotated with that concept. Semantic concepts are grouped into one of four semantic classes: (i) diseases from the National Library of Medicine (NLM) Medical Subject Headings (MeSH), (ii) human genes, (iii) SARS-CoV-2 proteins, and (iv) SARS-CoV-2 virus variants, plus additionally (v) a classification for long COVID. Preprints are also searchable using free text terms and Boolean operators.

**Fig. 1:**
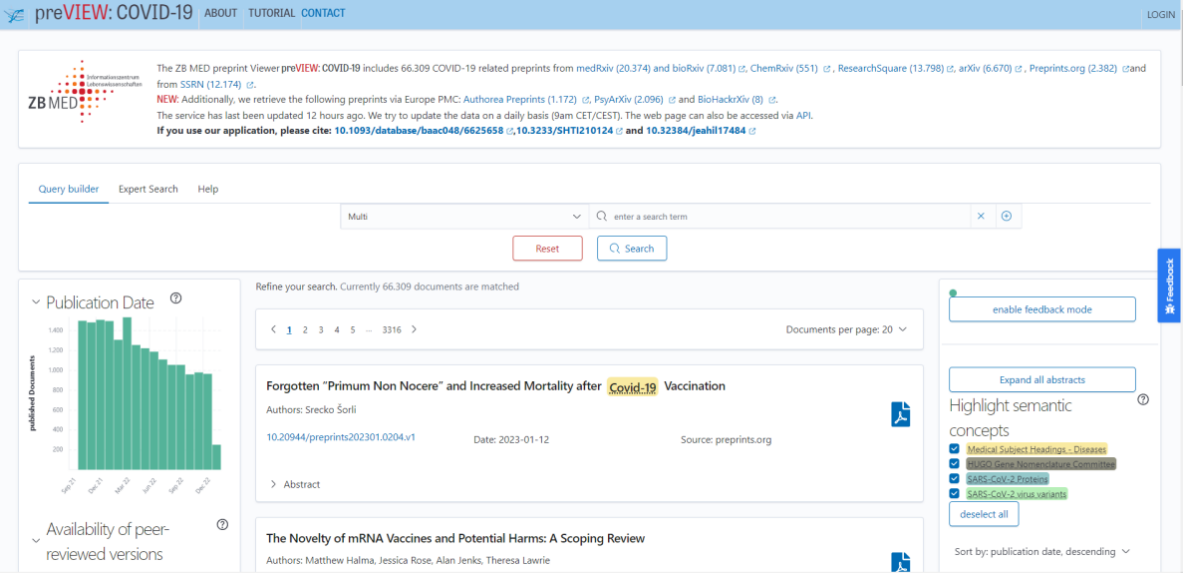
Screenshot of preVIEW main page.

**Fig. 2:**
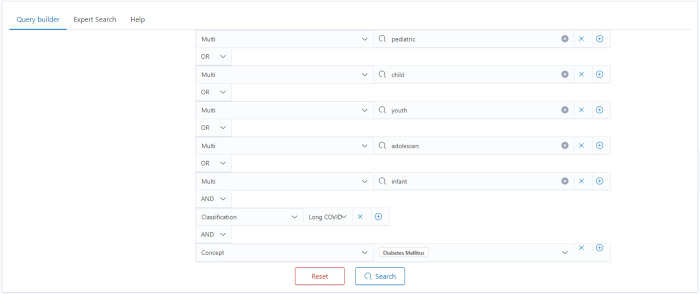
Example of a Query Builder search.

**Fig. 3:**
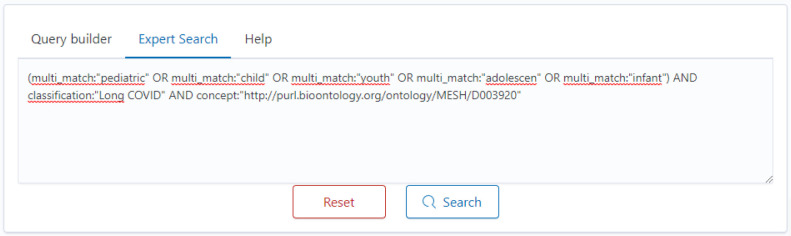
Example of an Expert search (the same search as Figure 2, manually entered).

Searches are constructed using the Query Builder or the Expert Search (Figure 1). In the Query Builder (Figure 2), choose which field to search (Title, Abstract, Concept, Date, or Multi), select parameters or type free text search terms depending on the field, and combine multiple lines using Boolean AND, OR, or NOT. Boolean operators cannot be used within each field line; use the Expert Search for more complex searches. In Expert Search (Figure 3), you can modify your most recent Query Builder search or write a query manually. You will need to use identifiers to search for semantic concepts, which are found by clicking on highlighted concepts in the results, or by modifying a Query Builder search. Expert Search also allows nested parentheses.

For both Query Builder and Expert Search, multiple terms, e.g., *loss of smell*, are searched as a phrase. End truncation is automatic in the free text fields – searching for either *inflammat* or inflammat* gives the same number of results.

### Results and limiters

Search results are sortable by publication date or relevance, and abstracts beneath each result can be expanded individually, or all toggled expanded or closed together. Side bars provide additional limits with changes reflected in the strategy box as well as in the results.

Results can be limited to preprints with a peer-reviewed, published version sourced from either Crossref, the preprint server, or preVIEW’s “Pre2Pub algorithm that automatically searches for a peer-reviewed article in PubMed” with a precision of 99.27% [1, 5]. Links to the peer-reviewed source are valuable as preprint servers often do not have the link, the published article’s DOI isn’t the same as the preprint DOI [6, 7], and the final title or authors may have changed.

Limits also include frequently occurring semantic concepts (a search for *mental health* in the multi field, will list Anxiety Disorders; Mental Disorders; and others under *MeSH Diseases*) and selecting any of these quickly narrows a search.

**Fig. 4:**
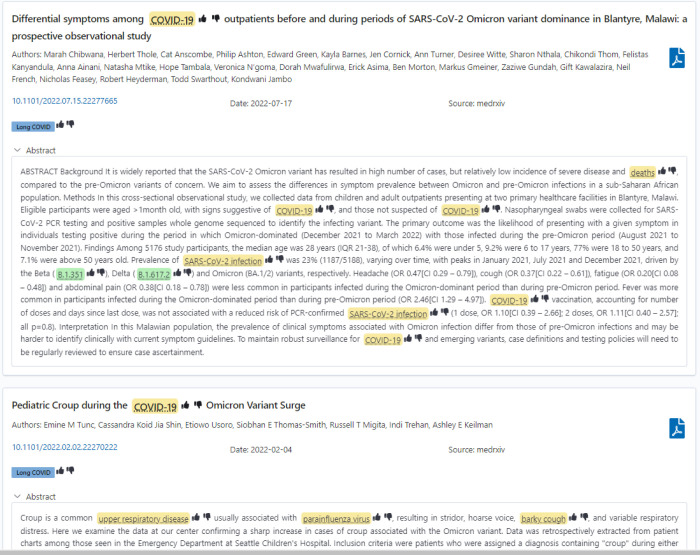
Example of preVIEW results with feedback mode enabled.

Semantic concepts are colour-coded in the result titles and abstracts. Clicking on any of the highlighted semantic concepts (e.g., omicron), will pop-up the preferred term (B.1.1.529) and further ontological information like scope notes and source. Users can provide feedback on any entry term by enabling the annotations feedback mode and clicking on a thumbs up or thumbs down (Figure 4).

### Data export

Results can be exported in Endnote format (.enw), RIS format (.ris), or BibTex (.bib), or by API. PreVIEW has a public RESTful type API: for instructions, email contact-preview@zbmed.de.

## Strengths

PreVIEW is most useful for COVID-19 searches on one of its semantic classes or long COVID. Preprints have already been restricted to COVID-19, it provides good flexibility for free text searching, and it’s extremely helpful that preVIEW checks for peer-reviewed final publications. The interface is intuitive, and the colour coding is helpful to skim for relevant results. I am glad to see long Covid as a preVIEW classification because the many synonyms for long COVID make it challenging to search.

## Weaknesses

PreVIEW results can only be downloaded as an entire set. This is a strong disadvantage for those who prefer to screen from the web interface and make selections, such as for informal or iterative searches, and they would have to click through to preprint sources one by one. The developers are very open to feedback, and I provided suggestions, so new export features may be added in future.

There are some errors that may be temporary. Using the NOT field tended to cause an error. On mobile (smartphone) browsers, parts of preVIEW’s display were squished, making it impractical to use.

PreVIEW does not save strategies or send alerts – your best option is to switch to Expert Search and copy the strings for your final search to a separate document.

As the developers state, preVIEW’s modular design will facilitate adding more semantic classes in future [4]. I would be interested in the addition of study designs (e.g., to narrow to systematic review preprints, or exclude case reports) and whether AI could successfully recognize more complex categories, such as COVID-19 patients versus non-COVID patients studied during the coronavirus pandemic. It would also be useful to limit by publication language.

## Comparison with Similar Products

### Europe PMC

Europe PMC (Europe PubMed Central https://europepmc.org/) has a vast number of preprints (530,000+ as of January 2023), has more preprint servers [8] than preVIEW, and is beginning to add text mining annotations [9]. A comparison of the number of COVID-19 preprints between Europe PMC and preVIEW finds 64,000 COVID-19 preprints in Europe PMC compared to over 66,000 in preVIEW. Europe PMC is fully functional on mobile browsers, provides more data export formats including XML and CSV, and offers valuable features such as limiting by publication language, exporting selections of results, saving searches, and sending email alerts.

PreVIEW’s strength compared to Europe PMC is the ability to quickly find all preprints on COVID-19 and preVIEW’s semantic concepts. Though Europe PMC has a keyword field that includes MeSH, this is not populated for preprints. Currently, Europe PMC doesn’t have an interface to search its new text mining annotations other than searching for if they are present.

### OSF Preprints

OSF Preprints (https://osf.io/preprints/) is another preprint aggregator with 2.3 million preprints and the ability to search using Boolean operators, truncation, and by field (like date or author keyword). However, there are no handy limits – limiting by COVID-19, date, or language must be written in the search string – and results cannot exported as a batch except by API.

### Medline, Embase, Cochrane CENTRAL

Comparing to broader bibliographic databases, all of Medline (through PubMed [10], LitCovid, or other platforms), Embase, and Cochrane CENTRAL have fewer preprints than preVIEW. Embase has the most preprints with just under 50,000 and less than 7,000 on COVID-19. Where preprints are of key interest, a preprint-specific aggregator should be added rather than relying on traditional bibliographic databases.

### LitCovid

LitCovid is limited to COVID-19 publications and has AI annotations for diseases, genes, and more, using PubTator [11]. However, these annotated results must be searched and downloaded through PubTator which is a less friendly and interactive interface than preVIEW.

## Additional Considerations

When selecting any semantic search engine, I recommend both (a) evaluating if the semantic concepts are well matched with your search intentions (*semantic mapping*) and (b) gaining at least a basic understanding of how the specific AI has been trained. Preprints are a great publishing stage to employ AI as preprints are transitional and plentiful, but understanding is key especially for systematic reviews where poor AI term matches might cause poor recall or precision.

Consider whether the semantic concepts of the search engine are well matched to your needs: are the concepts provided too broad or narrow for your search topic, or non-existent? If this is the case and you must develop a traditional keyword search, you may prefer one of the other aggregated preprint search engines. Semantic concepts may not exist yet for rapidly emerging topics. For example, pediatric multisystem inflammatory disease (MIS-C) doesn’t have a MeSH (as of January 2023), only a PubMed supplementary concept [12]. As preVIEW relies on MeSH for diseases, searching for *multisystem inflammatory syndrome* auto-suggests *Cryopyrin-Associated Periodic Syndromes* (a hereditary inflammatory disease) instead, which is different. Plus, preVIEW does not have semantic concepts for age groups like pediatrics. For MIS-C in preVIEW, it is best to develop a traditional keyword search.

Also, consider that AI annotations are not perfect. Machine learning incorporates strengths but also knowledge gaps in the training set. Continuing the example, the MIS in MIS-C preprints is tagged as Mullerian-inhibiting substance (MIS), which is a mismatch. For the long COVID classification, however, the preVIEW developers have thoughtfully developed AI training sets. They included a good range of synonyms and large, human-screened training sets with positive and negative examples, all of which should lead to good accuracy [1].

## Conclusion

Preprints are of high interest for rapidly evolving COVID topics, and searching preprint aggregators, such as preVIEW, provides broader coverage than single servers or bibliographic databases. With its intuitive search engine, its flexible features of both semantic and traditional searching, and being free online, preVIEW is worth trying for your search and worth monitoring as it develops new features. I recommend preVIEW for rapidly searching topics covered by preVIEW’s semantic concepts, such as for rapid reviews. Yet for topics not covered by the semantic concepts, and likely for systematic reviews, Europe PMC is a stronger option. If the searcher has the skill to develop a free-text keyword search, Europe PMC has slightly more preprint servers, and more export and alert features.
